# Importance of Bilateral Hip Assessments in Unilateral Lower-Limb Amputees: A Retrospective Review Involving Older Veterans

**DOI:** 10.3390/jcm13144033

**Published:** 2024-07-10

**Authors:** Seong Jin, Chi Hwan An, Ho Yong Jeong, Woohwa Choi, Sun-Won Hong, Hoon Ki Song, Hyun Sung Kim, Yun Kyung Lee, Hyo Jung Kang, Dong-young Ahn, Hea-Eun Yang

**Affiliations:** 1 Department of Physical Medicine and Rehabilitation, Veterans Health Service Medical Center, Seoul 05368, Republic of Korea; 2 Prosthetic and Orthotic Center, Veterans Health Service Medical Center, Seoul 05368, Republic of Korea

**Keywords:** lower-limb amputation, osteoporosis, bone mineral density, discordance in BMD

## Abstract

**Background/Objectives:** This study aimed to evaluate bone mineral density (BMD) discordance and its implications in veterans with unilateral lower-limb amputation, emphasizing the need for comprehensive hip assessments. **Methods:** Data were collected from 84 male veterans, and BMD was measured using dual-energy X-ray absorptiometry (DXA) at the lumbar spine, intact hip, and amputated hip. **Results:** The T-scores for the lumbar spine, intact hip, and amputated hip were −0.27 ± 1.69, −0.25 ± 1.20, and −1.07 ± 1.33, respectively. Osteoporosis and osteopenia were present in 19% and 34.6% of patients, respectively. Osteopenia and osteoporosis were most prevalent in the hips on the amputated side (32.1% and 13.1%, respectively), followed by the lumbar spines (22.6% and 8.3%) and the hips on the intact side (17.9% and 2.4%). BMD discordance between the lumbar spine and hip was found in 47.6% of participants, while discordance between both hips was observed in 39.3%. Transfemoral amputees had significantly lower BMD at the amputated hip compared to transtibial amputees (−2.38 ± 1.72 vs. −0.87 ± 1.16, *p* < 0.001). **Conclusions:** Veterans with unilateral lower-limb amputation exhibit a high prevalence of osteoporosis and significant BMD discordance, particularly between both hips. These findings underscore the necessity for bilateral hip assessments to ensure the accurate diagnosis and effective management of osteoporosis in this population.

## 1. Introduction

Reports from several studies have indicated that bone loss in the residual limb occurs in unilateral lower-limb amputees [1,2]. Reasons for low bone density on the amputated side include unloading and disuse for a certain period before and after amputation surgery, altered bone turnover, and changes in load distribution on both lower limbs while walking with a prosthesis [3,4,5]. Specifically, weight-bearing through a prosthetic socket offloads the distal femur, causing large areas of the femoral shaft and neck to experience significantly reduced levels of stimulation compared to weight-bearing on a healthy limb [6,7]. Bone loss occurs rapidly after lower-limb amputation and is not restored even up to 12 months after becoming ambulatory [8]. Bone density remains low in veterans with lower-limb amputation [9]. Patients with unilateral lower-limb amputation have a high risk of falling and are vulnerable to fractures due to decreased bone density on the amputated side; thus, the evaluation of bone mineral density and timely intervention are essential [10,11,12].

Osteoporosis is a well-known systemic skeletal condition that leads to an increased risk of fragility fractures [13]. However, two important facts about osteoporosis are commonly overlooked, especially in veterans with lower-limb amputation: osteoporosis is more likely to be underdiagnosed in men than women, and insufficient concern has been raised about discordance in bone mineral density (BMD) from both hips.

Since osteoporosis is more pronounced in postmenopausal women, men are relatively less evaluated for osteoporosis than women [14]. However, the prevalence of osteoporosis in men is also very high, reaching 12–20% depending on the study [15,16]. Additionally, when osteoporotic fractures occur, morbidity and mortality due to fractures are higher in men than in women [17,18]. The mortality rate during hospitalization for hip fracture is 10% for men and 5% for women, and within 1 year of discharge, the mortality rate is 38% for men and 28% for women [19]. Male sex itself is an independent factor associated with mortality following hip fracture [20]. Despite this, only about 10% of men with osteoporosis receive proper treatment [14]. 

Dual-energy X-ray absorptiometry (DXA) is a validated method for the determination of BMD and the gold standard for diagnosing osteoporosis [21]. DXA is usually measured at two central fracture-prone skeletal regions (spine and hip) and is determined by the lowest score [22,23]. When there is a discrepancy in categories of T-scores (osteoporosis, osteopenia, and normal) in the two skeletal sites, it is defined as discordance in BMD [24,25]. The classification of discordance is divided into no discordance (concordance), minor discordance, and major discordance. Minor discordance is defined as normal at one site and osteopenia at the other or osteopenia at one site and osteoporosis at the other, while major discordance is defined as normal at one site and osteoporosis at the other site [26]. Discordance in BMD is clinically significant because it can lead to inaccurate fracture risk assessments if only one site is assessed. This can result in missed diagnoses of osteoporosis and inappropriate treatment. Recognizing BMD discordance ensures comprehensive evaluation, accurate diagnosis, tailored interventions, and effective fracture prevention, thereby improving overall patient management. Higher rates of discordance between bilateral hip BMD are expected in patients with lower-limb amputation, whose amputated side often shows lower BMD compared to that of non-amputees. McMenemy et al. showed that combat-related traumatic injuries result in localized BMD loss in amputees, particularly at the femoral neck of the amputated limb, due to altered loading patterns post-amputation [27]. To our knowledge, only a few studies have primarily evaluated the discordance of bilateral hip BMD in amputees [27].

The goal of this study was to determine the difference in BMD across various skeletal sites and evaluate discordance in BMD based on each region. We hypothesized that discordance between both hips would be significant in unilateral lower-limb amputees.

## 2. Methods

Data were collected from the Orthotics and Prosthetics Center at Veterans Health Service Medical Center located in Seoul, Korea. Patients who had prostheses fabricated at the center and had BMD measurements at the same hospital between 2019 and 2023 were included. Out of the 1335 patients initially screened, 113 met the inclusion criteria of veterans with lower-limb amputation who used prostheses, regardless of the cause of amputation.

The exclusion criteria included patients with bilateral amputation or minor below-foot amputations, those who did not have BMD measured in one or more areas of the spine and both hips, the absence of a recorded amputation date, the presence of diseases affecting BMD or metal implants in the hips, and those with a weight greater than 136 kg. After applying these criteria, the final sample consisted of 84 patients. Among these, no patients had knee disarticulation. Demographic, anthropometric, and BMD data collected from the Orthotics and Prosthetics Center at Veterans Health Service Medical Center located in Seoul, Korea, were retrospectively reviewed.

Bone density was measured with either a Hologic Discovery W (Hologic, Bedford, MA, USA) or GE Lunar Prodigy (GE Healthcare, Madison, WI, USA). The manufacturer’s database matched for age, weight, and ethnicity was used for determining T-scores at the total hip and PA L1–L4 lumbar spine. To examine the presence of discordance, diagnoses from each skeletal region were made and compared to each other.

Statistical analysis was performed using the SPSS 19.0 Statistical Package for the Social Sciences (IBM Corp., Armonk, NY, USA). We conducted between-group comparisons using the independent *t*-test, Mann–Whitney U test for one variable (intact side hip BMD of the transfemoral group) that is not normally distributed, chi-square test, and Fisher’s exact test. Statistical significance was set at *p* < 0.05.

This study was conducted in accordance with the Declaration of Helsinki and approved by the Institutional Review Board of VHS Medical Center (IRB File No. 2023-12-016).

## 3. Results

### 3.1. Demographic Details and BMD Results

The general characteristics of the participants, all of whom were men, are described in Table 1. The T-scores of the lumbar spine, intact side hip, and amputated side hip were −0.27 ± 1.69, −0.25 ± 1.20, and −1.07 ± 1.33, respectively, with the amputated side hip exhibiting the lowest value. Osteoporosis was confirmed in 19% and osteopenia in 34.6% of the participants, according to the World Health Organization criteria for diagnosing osteoporosis using BMD.

The specific incidences of osteopenia and osteoporosis in different skeletal regions were analyzed. Osteopenia was observed in 32.1% of the hips on the amputated side, 17.9% of the hips on the intact side, and 22.6% of the lumbar spines. Osteoporosis was present in 13.1% of the hips on the amputated side, 2.4% of the hips on the intact side, and 8.3% of the lumbar spines.

### 3.2. Discordance between Different Skeletal Regions

Diagnosis based on the T-score of each region was compared to assess discordance in BMD. Discordances between T-scores in the lumbar spine and the hip are shown in Table 2. The results showed that 52.4% of the participants had T-score concordance between the spine and the hip, 41.6% had minor discordance, and 6.0% had major discordance. When assessed by region, 21.4% of participants had lower BMD in the hip, while 26.2% had lower BMD in the spine. 

The types of discordance between both sides of the hip are shown in Table 3. A total of 60.7% of the participants had T-score concordance between both sides, 28.6% had minor discordance, and 10.7% had major discordance. Based on sides, 34.5% of the patients had lower BMD on the amputated side, and 4.8% had lower BMD on the intact side. 

### 3.3. Impact of Level and Cause of Amputation on BMD

There were 11 transfemoral (TF) amputees and 73 transtibial (TT) amputees, and their mean T-scores of the amputated side hip were −2.38 ± 1.72 and −0.87 ± 1.16, respectively; this difference was statistically significant. Patient characteristics and BMD results according to the level of amputation are shown in Table 4. 

We sought to determine whether there were any differences depending on the cause of amputation. Seventeen patients were excluded because the cause could not be determined, fifty-six patients were confirmed to have traumatic causes, and eleven patients were confirmed to have vascular causes of amputation. In the case of traumatic amputation, the average time after amputation to BMD evaluation was long (43 years), whereas in the case of vascular amputation, the average time after amputation to BMD evaluation was relatively short (4.7 years). Consequently, the ages at which the BMD was assessed were 69 and 70 years in the traumatic causes and vascular causes groups, respectively, showing no significant difference. The mean T-score of the amputated side hip was lower in the traumatic amputation group than in the vascular amputation group (−1.25 ± 1.23 vs. −0.33 ± 1.68). Patient characteristics and bone mineral density results according to the cause of amputation are shown in Table 5. 

## 4. Discussion

### 4.1. Primary Outcomes

We have demonstrated that older veteran amputees had lower T-scores on the amputated side hip compared to the lumbar spine and intact side hip. Previous studies focusing on older amputees have reported reduced hip BMD in the amputated limb [28,29] and lower cortical density and area in the amputated limb compared to the intact limb due to insufficient load-bearing [1]. Smith et al. reported T-scores for the lumbar spine, sound side hip, and amputated side hip as −0.33 ± 1.89, −0.57 ± 1.15, and −1.38 ± 1.53, respectively, which are consistent with our results [28]. 

In a meta-analysis based on 86 studies performed in 2021, the prevalence of osteoporosis in the world was reported to be 18.3%. According to sex, the global prevalence of osteoporosis in women was reported to be 23.1%, while it was found to be 11.7% in men [16]. In our study, which included male veterans only, the prevalence of osteoporosis was substantially higher than the global prevalence of osteoporosis in men but lower than that in women.

The observed low bone density in veterans with lower-limb amputation and a high incidence of osteoporosis underscores the increased risk of fragility fractures in this population, emphasizing the importance of assessment and intervention to prevent falls and fractures.

The analysis revealed specific incidences of osteopenia and osteoporosis across different skeletal regions. Osteopenia was most prevalent in the hips on the amputated side (32.1%), followed by the lumbar spines (22.6%) and the hips on the intact side (17.9%). Similarly, osteoporosis was most prevalent in the hips on the amputated side (13.1%), followed by the lumbar spines (8.3%) and the hips on the intact side (2.4%). These findings indicate a higher prevalence of reduced bone density in the hips on the amputated side compared to other regions.

Our study also identified discordance in BMD between different skeletal sites, particularly between the lumbar spine and the hips, as well as between the intact and amputated sides of the hip. In previous studies on the discordance between the lumbar spine and the hip, the rate of discordance was highlighted as 41.7% (38.9% of minor discordance and 2.8% of major discordance) and 47.5% (43.3% of minor discordance and 4.2% of major discordance), as reported by Moayyeri et al. and Woodson et al., respectively [25,26]. Our results are in agreement with the results of previous studies regarding the discordance between the lumbar spine and the hip. 

Unlike discordance between the spine and the hip, discordance between both hips has been less studied. Our results revealed 39.3% of discordance (28.6% of minor discordance and 10.7% of major discordance) between both hips, and the T-score was lower in 87.7% of all cases with discordance, which is significant enough to warrant attention. Generally, five different causes have been proposed for the occurrence of discordance: physiologic, pathologic, anatomic, artifactual, and technical. Among them, discordance between the dominant hip and non-dominant hip is regarded as physiologic discordance, which is caused by differences in exposure to mechanical strain such as weight-bearing [25]. This type of discordance is known to be usually 10% or less [30,31]. Based on these studies, some argue that measuring hip BMD on both sides is unnecessary and that the measurement of a single femur is sufficient. This might be true in non-amputees; however, in individuals with major lower-limb amputation, BMD on the amputated side decreases and is more severe in TF amputations than in TT amputations [9,28]. 

Recent findings by McMenemy et al. support our observations, indicating that combat-related traumatic injuries are associated with localized BMD reduction in amputees, especially at the femoral neck of the amputated limb. This BMD loss is attributed to altered loading patterns post-amputation. Their findings suggest that the reduced BMD observed is more likely due to localized unloading osteopenia, driven by the prosthetic socket design and reduced mechanical stimulus rather than systemic factors [27]. These insights emphasize the importance of evaluating BMD in both hips for amputees to capture these biomechanical changes accurately.

Discordance in BMD poses challenges for fracture risk assessment and management, as discrepancies between skeletal sites may lead to inaccurate assessments of fracture risk. Particularly, in patients with unilateral lower-limb amputation, hip BMD must be measured on both sides to ensure an accurate diagnosis. Clinicians should be aware of the potential for discordance in BMD and consider multiple skeletal sites when evaluating fracture risk in patients with lower-limb amputation.

### 4.2. Secondary Outcome

Additionally, our study highlights the impact of the level of amputation on bone health outcomes. Patients with TF amputation exhibited significantly lower BMD compared to those with TT amputation, underscoring the importance of considering the level of amputation when assessing bone health in this population. In a retrospective cohort study that involved British male veterans with unilateral lower-limb amputation, there was a significant decrease in femoral neck BMD on the amputated side; the decrease was more prominent in TF amputees (T-score: −2.26) than in TT amputees (T-score: −1.10) [9]. The result was consistent with ours; notably, the scope of their study resembled ours in that it targeted older veterans with an average age of 73 years, but the number of participants was smaller at 44 in total. 

Differences depending on the cause of amputation were also analyzed. The T-score of the amputated side hip was lower in traumatic amputation compared to vascular amputation, but these findings should be interpreted cautiously. Patients with traumatic amputation had longer time intervals between amputation and BMD assessment compared to those with vascular amputation, suggesting that the amputation occurred at a much younger age in the traumatic amputation cohort. Of particular note here is the number of TF amputees in each group. As previously mentioned, the decrease in hip BMD was more severe in TF amputation than in TT amputation, but there was no single TF amputation among the vascular amputation group in our study. There are limitations in the determination of the level of amputation in traumatic amputation, but in the case of vascular amputation, distal amputation is selected, if possible, which is advantageous in terms of recovery and function [32]. Advancements in revascularization approaches and the establishment of integrated wound care contributed to the decreased rates of proximal amputation in patients with lower-extremity vascular disease [33]. This might explain why there was no TF amputation among the vascular amputation group, potentially biasing the result.

### 4.3. Limitations

Certain limitations exist in this study. It is a retrospective study without age-matched control. Additionally, information regarding the prostheses worn by the participants was not considered in the analyses due to the complexity and difficulties with standardization. Moreover, information about the participants’ ambulatory function, prosthesis use time, K-levels, and results from the 6-Minute Walk Test or Timed-Up-and-Go test were lacking. This absence of activity level data may impact the understanding of the relationship between physical activity and BMD in the amputated limb.

Large differences in sample sizes between subgroup comparisons could be another limitation of our study. Nevertheless, we believe that our study presents important information regarding the changes in BMD that appear in amputated veterans. Future research on the general population could help establish a more general consensus.

## 5. Conclusions

In conclusion, our study provides valuable insights into bone health outcomes in veterans with unilateral lower-limb amputation, revealing a high prevalence of osteoporosis, discordance in BMD between skeletal sites, and associations with the level and cause of amputation. In patients with unilateral lower-limb amputation, the BMD of the amputated side hip was lower than that of the intact side hip, and discordance in BMD between both sides was observed in more than one-third of the study sample. The BMD of the amputated side hip is lower in TF amputation than in TT amputation. These findings emphasize the importance of the simultaneous examination of both hips in patients with lower-limb amputation, especially in those who had TF amputation.

## Figures and Tables

**Table 1:** Patient Characteristics (*n* = 84).

Characteristic	Mean ± SD/*n* (%)
Age	69.0 ± 8.6
Time after amputation (years)	38.4 ± 18.5
Amputation side (right/left)	43 (51.2%):41 (48.8%)
Amputation level (transfemoral/transtibial)	11 (13.1%):73 (86.9%)
Height (cm)	165.9 ± 5.7
Weight (kg)	65.2 ± 9.0
Body mass index	23.7 ± 2.7
T-score of lumbar spine	−0.27 ± 1.69
T-score of intact side hip	−0.25 ± 1.20
T-score of amputated side hip	−1.07 ± 1.33
Normal/Osteopenia/Osteoporosis	39 (46.4%):29 (34.6%):16 (19.0%)

**Table 2:** Discordance in BMD between the Spine and the Hip (*n* = 84).

		Number of Patients	Observed Prevalence (%)
Concordance		44	52.4
Minor discordance	Lower in hip	16	19.0
	Lower in spine	19	22.6
Major discordance	Lower in hip	2	2.4
	Lower in spine	3	3.6

BMD: bone mineral density.

**Table 3:** Discordance in BMD between Both Hips (*n* = 84).

		Number of Patients	Observed Prevalence (%)
Concordance		51	60.7
Minor discordance	Lower in amputated side	22	26.2
	Lower in intact side	2	2.4
Major discordance	Lower in amputated side	7	8.3
	Lower in intact side	2	2.4

BMD: bone mineral density.

**Table 4:** Patient Characteristics and T-Scores According to the Level of Amputation.

	Transfemoral (*n* = 11)	Transtibial (*n* = 73)	*p*-Value
Age	70.4 ± 9.3	68.8 ± 8.6	0.570
Time after amputation (years)	46.6 ± 11.0	37.3 ± 19.1	0.160
Height (cm)	165.1 ± 5.9	166.0 ± 5.6	0.659
Weight (kg)	61.3 ± 8.3	65.9 ± 9.0	0.124
Body mass index	22.4 ± 2.3	23.9 ± 2.7	0.099
T-score of lumbar spine	−0.84 ± 1.57	−0.19 ± 1.70	0.237
T-score of amputated side hip	−2.38 ± 1.72	−0.87 ± 1.16	0.000 *
T-score of intact side hip	−0.79 ± 1.87	−0.17 ± 1.06	0.111

Values are presented as means ± standard deviations. * Statistical significance.

**Table 5:** Patient Characteristics and T-Scores According to the Cause of Amputation.

	Trauma (*n* = 56)	Vascular (*n* = 11)	*p*-Value
Age	69.4 ± 9.0	70.8 ± 5.6	0.606
Time after amputation (years)	43.4 ± 14.2	4.7 ± 7.7	0.000 *
Transfemoral/transtibial	10:46	0:11	0.195
Height (cm)	166.1 ± 5.8	165.4 ± 6.1	0.734
Weight (kg)	66.4 ± 9.6	62.4 ± 6.7	0.215
Body mass index	24.0 ± 2.7	22.7 ± 1.5	0.160
T-score of lumbar spine	−0.32 ± 1.55	−0.07 ± 1.10	0.610
T-score of amputated side hip	−1.25 ± 1.23	−0.33 ± 1.68	0.036 *
T-score of intact side hip	−0.31 ± 1.14	−0.26 ± 1.52	0.881

Values are presented as means ± standard deviations. * Statistical significance.

## Data Availability

The data utilized and examined in this study are detailed within the article, and further inquiries can be directed to the corresponding author upon reasonable request.

## References

[1] Sherk V.D., Bemben M.G., Bemben D.A. (2008). BMD and bone geometry in transtibial and transfemoral amputees. J. Bone Miner. Res..

[2] Leclercq M.M., Bonidan O., Haaby E., Pierrejean C., Sengler J. (2003). Study of bone mass with dual energy x-ray absorptiometry in a population of 99 lower limb amputees. Ann. Readapt. Med. Phys..

[3] van Velzen A.D., Nederhand M.J., Emmelot C.H., Ijzerman M.J. (2005). Early treatment of trans-tibial amputees: Retrospective analysis of early fitting and elastic bandaging. Prosthet. Orthot. Int..

[4] Esquenazi A., DiGiacomo R. (2001). Rehabilitation after amputation. J. Am. Podiatr. Med. Assoc..

[5] Davies B., Datta D. (2003). Mobility outcome following unilateral lower limb amputation. Prosthet. Orthot. Int..

[6] Kaufmann J.J., McMenemy L., Phillips A.T.M., McGregor A.H., Bull A.M.J., Clasper J., Mahoney P.F. (2022). Bone health in lower-limb amputees. Blast Injury Science and Engineering: A Guide for Clinicians and Researchers.

[7] Gailey R., Allen K., Castles J., Kucharik J., Roeder M. (2008). Review of secondary physical conditions associated with lower-limb amputation and long-term prosthesis use. J. Rehabil. Res. Dev..

[8] Bemben D.A., Sherk V.D., Ertl W.J.J., Bemben M.G. (2017). Acute bone changes after lower limb amputation resulting from traumatic injury. Osteoporos. Int..

[9] Kulkarni J., Adams J., Thomas E., Silman A. (1998). Association between amputation, arthritis and osteopenia in British male war veterans with major lower limb amputations. Clin. Rehabil..

[10] Iosa M., Paradisi F., Brunelli S., Delussu A.S., Pellegrini R., Zenardi D., Paolucci S., Traballesi M. (2014). Assessment of gait stability, harmony, and symmetry in subjects with lower-limb amputation evaluated by trunk accelerations. J. Rehabil. Res. Dev..

[11] Hunter S.W., Batchelor F., Hill K.D., Hill A.M., Mackintosh S., Payne M. (2017). Risk factors for falls in people with a lower limb amputation: A systematic review. PM R.

[12] Kulkarni J., Wright S., Toole C., Morris J., Hirons R. (1996). Falls in patients with lower limb amputations: Prevalence and contributing factors. Physiotherapy.

[13] LeBoff M.S., Greenspan S.L., Insogna K.L., Lewiecki E.M., Saag K.G., Singer A.J., Siris E.S. (2022). The clinician’s guide to prevention and treatment of osteoporosis. Osteoporos. Int..

[14] Bandeira L., Silva B.C., Bilezikian J.P. (2022). Male osteoporosis. Arch. Endocrinol. Metab..

[15] Zamani M., Zamani V., Heidari B., Parsian H., Esmaeilnejad-Ganji S.M. (2018). Prevalence of osteoporosis with the World Health Organization diagnostic criteria in the Eastern Mediterranean Region: A systematic review and meta-analysis. Arch. Osteoporos..

[16] Salari N., Ghasemi H., Mohammadi L., Behzadi M.H., Rabieenia E., Shohaimi S., Mohammadi M. (2021). The global prevalence of osteoporosis in the world: A comprehensive systematic review and meta-analysis. J. Orthop. Surg. Res..

[17] Vilaca T., Eastell R., Schini M. (2022). Osteoporosis in men. Lancet Diabetes Endocrinol..

[18] Bliuc D., Nguyen N.D., Milch V.E., Nguyen T.V., Eisman J.A., Center J.R. (2009). Mortality risk associated with low-trauma osteoporotic fracture and subsequent fracture in men and women. JAMA.

[19] Jiang H.X., Majumdar S.R., Dick D.A., Moreau M., Raso J., Otto D.D., Johnston D.W. (2005). Development and initial validation of a risk score for predicting in-hospital and 1-year mortality in patients with hip fractures. J. Bone Miner. Res..

[20] Haentjens P., Magaziner J., Colon-Emeric C.S., Vanderschueren D., Milisen K., Velkeniers B., Boonen S. (2010). Meta-analysis: Excess mortality after hip fracture among older women and men. Ann. Intern. Med..

[21] Blake G.M., Fogelman I. (2007). The role of DXA bone density scans in the diagnosis and treatment of osteoporosis. Postgrad. Med. J..

[22] Yoon B.H., Kim D.Y. (2021). Discordance between Hip and Spine Bone Mineral Density: A Point of Care. J. Bone Metab..

[23] Kanis J.A., Harvey N.C., McCloskey E., Bruyere O., Veronese N., Lorentzon M., Cooper C., Rizzoli R., Adib G., Al-Daghri N. (2020). Algorithm for the management of patients at low, high and very high risk of osteoporotic fractures. Osteoporos. Int..

[24] Faulkner K.G., von Stetten E., Miller P. (1999). Discordance in patient classification using T-scores. J. Clin. Densitom..

[25] Woodson G. (2000). Dual X-ray absorptiometry T-score concordance and discordance between the hip and spine measurement sites. J. Clin. Densitom..

[26] Moayyeri A., Soltani A., Tabari N.K., Sadatsafavi M., Hossein-Neghad A., Larijani B. (2005). Discordance in diagnosis of osteoporosis using spine and hip bone densitometry. BMC Endocr. Disord..

[27] McMenemy L., Behan F.P., Kaufmann J., Cain D., Bennett A.N., Boos C.J., Fear N.T., Cullinan P., Bull A.M.J., Phillips A.T.M. (2023). Association between combat-related traumatic injury and skeletal health: Bone mineral density loss is localized and correlates with altered loading in amputees: The Armed Services Trauma Rehabilitation Outcome (ADVANCE) Study. J. Bone Miner. Res..

[28] Smith É., Comiskey C., Carroll Á., Ryall N. (2011). A study of bone mineral density in lower limb amputees at a national prosthetics center. JPO J. Prosthet. Orthot..

[29] Rush P.J., Wong J.S., Kirsh J., Devlin M. (1994). Osteopenia in patients with above knee amputation. Arch. Phys. Med. Rehabil..

[30] Faulkner K.G., Genant H.K., McClung M. (1995). Bilateral comparison of femoral bone density and hip axis length from single and fan beam DXA scans. Calcif. Tissue Int..

[31] Bonnick S.L., Nichols D.L., Sanborn C.F., Payne S.G., Moen S.M., Heiss C.J. (1996). Right and left proximal femur analyses: Is there a need to do both?. Calcif. Tissue Int..

[32] Crane H., Boam G., Carradice D., Vanicek N., Twiddy M., Smith G.E. (2021). Through-knee versus above-knee amputation for vascular and non-vascular major lower limb amputations. Cochrane Database Syst. Rev..

[33] Barnes J.A., Eid M.A., Creager M.A., Goodney P.P. (2020). Epidemiology and Risk of Amputation in Patients With Diabetes Mellitus and Peripheral Artery Disease. Arterioscler. Thromb. Vasc. Biol..

